# Voices from the classroom: Teachers’ perspectives on collaboration with speech-language therapists in learners with special educational needs schools

**DOI:** 10.4102/sajcd.v73i1.1169

**Published:** 2026-05-28

**Authors:** Haseena Mahomed Joosub, Marguerite De Jongh, Rahab Mothapo

**Affiliations:** 1 Department of Speech-Language Pathology and Audiology, Faculty of Health Sciences, Sefako Makgatho Health Sciences University, Pretoria, South Africa

**Keywords:** collaboration, disabilities, individual support plan, interdisciplinary collaborative model, learners with special educational needs, speech-language therapists, teachers, augmentative and alternative communication

## Abstract

**Background:**

Effective collaboration between teachers and speech-language therapists (SLTs) is crucial in supporting learners with special educational needs (LSEN). Despite its importance, research on collaboration as well as the role of the SLT in South African LSEN schools remains limited.

**Objectives:**

This study aimed to explore teachers’ understanding of the SLT’s role in LSEN schools, their experiences of working with SLTs, and their perceptions of the value of collaboration with SLTs in these settings.

**Method:**

An exploratory qualitative design was used. Semi-structured interviews with teachers from four LSEN schools in a district in South Africa were employed. Data were analysed using an inductive coding approach to identify key themes and patterns.

**Results:**

Teachers’ perspectives on collaboration with SLTs were both positive and negative. Positive outcomes included support to learners with augmentative and alternative communication (AAC), enhanced communication skills and classroom participation for learners, hearing assessments and tailored intervention strategies. Challenges cited included limited SLT availability because of competing demands and a shortage of SLTs in LSEN schools.

**Conclusion:**

In the South African context, strengthening interdisciplinary collaboration among SLTs, teachers, parents and other professionals is essential for improving learner outcomes in LSEN schools. Recommendations include structured communication, joint planning and targeted professional development to enhance collaborative practices.

**Contribution:**

This study contributes to the understanding of teacher–SLT collaboration within LSEN schools and how this may benefit LSEN.

## Introduction

Effective collaboration between professionals working with learners with special educational needs (LSEN) has gained global recognition. In South Africa, however, research on collaboration between teachers and speech-language therapists (SLTs), particularly within LSEN schools, remains limited.

Collaboration in education involves professionals such as SLTs and teachers working together, sharing expertise and establishing common goals to support learners (Brimo & Huffman, 2023). A shared commitment is crucial in addressing the varied needs of learners (Archibald, 2017; Mampane, 2016). Effective collaboration enhances communication, clarifies professional roles and improves the implementation of strategies, ultimately leading to better learner outcomes (Olszewski et al., 2019; Pentek et al., 2018). The South African Speech-Language-Hearing Association (SASLHA) emphasises collaboration as a key responsibility of SLTs working in educational settings.

Archibald (2017) emphasised that SLTs must work closely with teachers to identify learners’ barriers to learning and to provide appropriate therapeutic services (Geertsema & Le Roux, 2020). SLTs assess and intervene in communication and swallowing disorders across all age groups, and their role includes collaboration with other team members in educational settings, particularly within classrooms.

SLTs thus play a vital role in supporting LSEN (Dillon et al., 2021), especially within interdisciplinary teams that include teachers, occupational therapists, social workers, physiotherapists, counsellors and educational psychologists. This team-based approach ensures comprehensive service delivery (Mitchell et al., 2020). As observed in South African LSEN schools and supported by Curro et al. (2022), effective interdisciplinary collaboration remains essential for meeting the diverse needs of these learners.

Pfeiffer et al. (2019) reported that determining LSEN school eligibility for learners requires conducting comprehensive assessments and providing interventions which demand a collaborative team approach. South African LSEN schools utilise interdisciplinary assessments to gauge functional levels and identify challenges, necessitating collaboration to align goals, track progress and ensure learner-centred outcomes. Hence, this critical interdisciplinary model is vital for the academic and holistic development of LSEN.

Although interdisciplinary collaboration in education is increasingly acknowledged, limited research exists on teacher–SLT collaboration in both mainstream and LSEN schools in South Africa (Sisti & Robledo, 2021). Based on the gap observed by the researcher, this study explored teachers’ insights into working with SLTs in LSEN schools, focusing on their experiences of SLT roles, collaborative practices and the perceived value of these professional relationships.

The study was guided by the interdisciplinary collaborative model, which emphasises joint efforts in assessment, goal development and intervention planning to support learners holistically (Dillon et al., 2021). Interdisciplinary collaboration between SLTs and teachers is vital for overcoming barriers and enhancing educational experiences for LSEN (Mampane, 2016; Wium & Louw, 2015). This model, which has proven effective in LSEN settings, aligns with the study’s aim and highlights the broader impact of collaborative efforts on the educational system (Brimo & Huffman, 2023).

The aim of the study was to explore teachers’ perceptions of collaboration with SLTs in LSEN schools. The objectives that align with the aim, and are embedded within it, include:

- To explore teachers’ understanding of the role of SLTs in LSEN schools.
- To explore teachers’ experiences of working with SLTs.
- To determine teachers’ perceptions of the importance of collaboration with SLTs in LSEN schools.

## Research methods and design

### Study design

An exploratory qualitative approach was employed to gain insight into teachers’ perceptions of collaboration with SLTs and to capture variations in individual experiences (Allsop et al., 2022; Hashmi et al., 2017). This approach enriched the understanding of collaborative dynamics within LSEN schools.

### Setting

The study was conducted in four LSEN schools located in the Tshwane South District, South Africa. These schools cater to learners aged 6 years to 18 years with a range of disabilities, including intellectual and physical disabilities, autism spectrum disorder (ASD), specific learning disabilities, deafness, attention-deficit and hyperactivity disorder (ADHD), epilepsy and visual impairments. Learners with ASD are, however, accommodated until the age of 21 years.

### Study population and sampling strategy

Teachers from the four LSEN schools were purposively selected based on shared characteristics and specific inclusion criteria. These included collaborating with SLTs; having SLT-supported learners in their classrooms; being registered with the South African Council for Educators (SACE), providing informed consent (Etikan et al., 2016) and working with LSEN aged 7–11 years (foundation phase). Under the *South African Schools Act No. 84 of 1996*, a uniform national system for school management and finance was established. This legislation notably mandates compulsory primary schooling for all children starting at age seven years until they reach fifteen years (Van Rensburg et al., 2024). This is the phase that is usually prioritised for therapy and support as reported by De Villiers (2012). The early childhood phase is widely recognised by diverse research fields as fundamental for lifelong learning, despite often presenting challenges such as Specific Learning Difficulties (SpLD). Therefore, it is vital to detect and prevent potential SpLDs (and/or other disorders) during this significant developmental period. Once ethical approval was granted, the School-Based Support Team (SBST) managed the recruitment of participants. Drawing on their comprehensive understanding of school-wide challenges and their regular oversight of teacher and learner development (Nong, 2020), the SBST was able to identify and select individuals most suited for the study’s objectives. To minimise bias, participants were selected from similar LSEN schools that met the study criteria (Smith & Noble, 2014).

### Data collection

A pilot study was conducted with teachers from other LSEN schools to confirm the effectiveness of the data collection tools and procedures.

Data collection was conducted in two phases to explore teachers’ perceptions and experiences of collaboration with SLTs in LSEN schools.

In Phase 1, 21 participants completed demographic questionnaires that captured information on ethnicity, age, gender, professional training and experience working with LSEN. The aim of the questionnaire was to obtain a foundational understanding of the study population and quantitative data on their experience working in LSEN schools and inform the subsequent interview phase.

Phase 2, involved in-depth, semi-structured interviews with 11 participants. The interview schedule was adapted from previous studies (Archibald, 2017; Baxter et al., 2009; Pentek et al., 2018; Van Wyk, 2020) and included open-ended questions designed to elicit detailed responses about collaboration with SLTs. Follow-up questions were used to probe deeper into participants’ experiences. The interviews were conducted in English because it is the language of learning and teaching (LoLT) at the LSEN schools included in the study. Furthermore, Mkhize and Balfour (2017) considered English to be the primary language in which learning, teaching and research take place in South Africa.

Data collection through semi-structured interviews continued until data saturation was reached, ensuring that no new themes emerged as has been done in another study (Hennink & Kaiser, 2022). This phase allowed for a rich exploration of the collaborative dynamics between teachers and SLTs in LSEN settings. Validity was supported by allowing sufficient time for responses and through member checking after each interview (Noble & Smith, 2015). Internal validity was also ensured by securely storing the results and reviewing the results time and again to determine whether there was consistency across responses (Lin, 2009).

### Data analysis

Phase 1 involved descriptive statistics to summarise participants’ demographic and background information (Kaur et al., 2018).

In Phase 2, interview data were transcribed using Happy Scribe™ software and analysed using Braun and Clarke’s six-step thematic analysis framework (2023), supported by Nowell et al. (2017). An inductive coding approach was used to allow themes and patterns to emerge from the data (Naeem et al., 2023). Microsoft Excel spreadsheet was used to organise and interpret relationships among concepts.

To ensure *dependability*, a peer reviewer verified the transcripts and coding. Two supervisors oversaw all stages of the analysis including validating the coding scheme to ensure consistency and rigour (Stahl & King, 2020).

*Confirmability* was maintained through transparent documentation (Gunawan, 2015), and *transferability* was supported by detailed descriptions of participants, settings and methods to aid contextual understanding (Stahl & King, 2020).

### Ethical considerations

Ethical clearance was obtained from the Sefako Makgatho University and School Ethics Committees (SMUREC/H/24/2023:PG). Participants provided written informed consent after being briefed on the study’s purpose, procedures, confidentiality and their right to withdraw at any time.

Confidentiality was maintained in accordance with the *Protection of Personal Information Act* (Adams et al., 2021). All data were securely stored on a password-protected Google Drive, accessible only to the researcher and supervisors.

## Results

### Participant characteristics – Phase 1

The participants’ characteristics encompassed key demographic and professional details, including their age, the nature of their training to teach LSEN, their years of experience in LSEN education, and the degree to which they engaged in collaborative practices with SLTs and other interdisciplinary team members. These attributes provided valuable context for understanding the perspectives and experiences shared during the study. Table 1 outlines the demographic profile of the 21 participants included in Phase 1, offering a foundation for interpreting the qualitative data that emerged in Phase 2.

**TABLE 1:** Participant information.

Variable	Participants
**Ethnic group**	
Participants working with LSEN represented diverse racial backgrounds, including white, black and Indian people.	The sample (*n* = 21) comprised 12 white educators (57%), eight black educators (38%) and one Indian educator (5%) reflecting the demographic profile of staff in the selected LSEN schools.
**Gender**	
The participants consisted of both male and female.	The participant cohort featured 20 female respondents (95%) and one male respondent (5%).
**Age**	
The ages of the 21 participants were categorised into four groups: 20–30, 31–40, 41–50 years and a senior cohort of 50 years and above.	The study included diverse age groups, with the largest representation found in the 31–40 year category (38%, *n* = 8). Participants aged 50 years and above accounted for 29% (*n* = 6), followed by the 20–30 years age group (24%, *n* = 5) and those between 41 years and 50 years (9%, *n* = 2).
**Training of educators (Working with LSEN)**	
Participants’ readiness to work with LSEN ranged from having no training, to receiving partial informal training through workshops, to completing full formal training programmes.	Five participants (24%) had received no formal or informal training, eight participants (38%) were partially trained, and eight participants (38%) were fully trained in working with LSEN.
**Years of work experience in LSEN schools**	
Participants reported LSEN school experience spanning from 1 year to 25 years. These were grouped into 5-year brackets (1–5, 6–10, 11–15, 16–20 and 21–25 years) to determine if the significance of collaboration is perceived differently by novice versus experienced teachers.	Data showed participants with 6–10 years (48%, *n* = 10), 11–15 years (19%, *n* = 4) and 21–25 years (5%, *n* = 1) of service. Early-career educators with 1–5 years of experience comprised the remaining 28% (*n* = 6).

LSEN, learners with special educational needs.

Table 1 presents a demographic summary of Phase 1 (questionnaires) of participants’ information, detailing their ethnicity, age, gender, special needs training received and years of relevant work experience.

### Interview results – Phase 2

Phase 2 consisted of qualitative data gathered from one-on-one interviews. The following themes and codes (Table 2) emerged from the raw data.

**TABLE 2:** Themes and codes.

Themes	Codes
SLT support	Hearing intervention Augmentative and alternative communication modes Speech and language intervention Emotional support for learners Individual and group therapy
Enhanced team collaboration	Remedial therapy Academic support Nursing support Occupational Therapy support Psychological support Social work support
Effect of team collaboration	Improvement in children’s communication Consulting with parents Adherence to medication No benefits Gross motor improvement Feeding and swallowing skills improvement Placement back into mainstream education
Collaborative planning and monitoring	Learner benefits Planning activities Unified vision and mission
Guidance and intervention	Improved classroom participation Speech production Classroom activities Advice on medical background (of learners).
Collaborative communication methods	Face-to-face exclusively A combination of face-to-face, WhatsApp and the Antecedent/Behaviour/Comment (ABC) written system. Formal and informal meetings Consultations and parent meetings
Collaborative involvement	Limited involvement Functional involvement
Collaborative relationship	Poor relationship Poor communication

SLT, speech-language therapist.

Table 2 provides a tabulated summary of the themes and their corresponding codes. Themes represent prevalent topics in participant responses, while codes denote the subtopics identified within each theme.

Furthermore, Table 3 provides a succinct overview of the identified themes and their associated codes, thereby elucidating the study’s core findings.

**TABLE 3:** Themes, description of results and codes.

Themes	Description of results	Codes
The role of SLTs in LSEN schools	Participants reported that SLTs at their schools enhanced learners’ communication skills and classroom participation through AAC and speech-language therapy, in addition to conducting hearing assessments, interventions and guiding classroom activities.	Hearing intervention Augmentative and alternative communication modes Speech and language intervention Emotional support for learners Individual and group therapy
Collaboration benefits and practices	Participants emphasised numerous advantages of interdisciplinary team collaboration, noting its role in enhancing learners’ communication, facilitating parent consultations and ensuring medication adherence, all contributing to improved academic performance. Collaborative support included academic, nursing, occupational therapy, psychological and social work. Observed improvements included communication, gross motor skills, feeding and swallowing and medication adherence, potentially aiding mainstream.	Improvement in children’s communication Consulting with parents Adherence to medication No benefits Gross motor improvement Feeding and swallowing skills improvement Placement back into mainstream education
Teachers’ views on collaborating with SLTs	Participants presented a mixed view on SLT involvement where positive, functional engagement and limited involvement of SLTs was noted. Participants attributed this lack of support to SLTs’ extensive extra duties beyond their scope. Another point reinforced and highlighted was the scarcity of SLTs in LSEN schools. Addressing these issues is crucial for fostering a collaborative school environment, with participants offering solutions. Conversely, some participants experienced no challenges.	Extra commitments Inadequate support Limited number of SLTs No challenges Inconsistent SLT allocations Intrusion in classrooms Inconsiderate schedule allocation Restricted scope Poor communication within SLT department
Teacher–SLT Collaboration Needs	To foster a positive collaborative platform in schools, participants suggested key solutions: emphasising training for teachers on SLT roles and collaborative importance to enhance service utilisation and advocating for improved SLT–teacher relationships. Furthermore, they recommended reducing SLTs’ extraneous duties, promoting therapist retention for learner rapport and increasing SLT employment in LSEN schools to meet the demands.	Teacher training Improved teacher support Developing relationships between teacher and SLT Reduce other commitments and administrative duties of SLTs Retain therapists in their current working allocations Advise on and provide adaptive resources Consider classroom schedules Increase therapy period Allocate more SLTs

LSEN, learners with special educational need; SLT, speech-language therapist; AAC, augmentative and alternative communication.

Table 3 illustrates what participants conveyed through their specific codes, which collectively point to the predominant themes. Henceforth, detailed findings from Phase 1 are discussed next, drawing connections to broader literature for a more evidence-based perspective.

## Discussion

### Phase 1

#### Training and experience of participants

Findings from the questionnaire revealed that 38.1% (*n* = 8) of participants reported to have received the necessary training to teach LSEN, while another 38.1% (*n* = 8) were only partially trained. Notably, 23.8% (*n* = 5) of teachers indicated that they had received no formal or informal training. This lack of training, particularly regarding collaboration and its importance in LSEN schools, has been highlighted in previous studies (Banks, 2018; Pentek et al., 2018; Pfeiffer et al., 2019). This presents significant challenges, acting as a barrier to effective teamwork and negatively impacting learner performance and progress (Pentek et al., 2018; Pfeiffer et al., 2019).

In terms of experience, 47.6% (*n* = 10) of participants had between 6 years and 10 years of experience working in LSEN schools, 28.6% (*n* = 6) had 1 year to 5 years, and 19% (*n* = 4) had 11 years or more years of experience.

Professional experience does not appear to mitigate the fundamental issue of inadequate pre-service training in collaborative practice. The sentiment articulated in Brimo and Huffman’s (2023) study, where participants noted they haven’t been taught to have knowledge of collaboration’ (Brimo & Huffman, 2023) suggests that this deficiency spans teachers at various stages of their careers. This underscores the need for targeted training on interdisciplinary collaboration, which has significant implications for service provision in LSEN schools.

#### Awareness and engagement in collaboration

Most participants (86%, *n* = 18) reported being aware of collaborative practices at their schools, and 90% (*n* = 19) indicated active engagement in collaboration with SLTs and other team members. This reflects a positive trend towards interdisciplinary collaboration in LSEN settings.

The frequency of collaboration among participants varied considerably. A notable proportion, 43% (*n* = 9), reported engaging in collaborative practices with SLTs and other team members on a weekly basis. In contrast, 19% (*n* = 4) indicated that collaboration occurred monthly, while another 19% (*n* = 4) reported daily collaboration.

Although literature offers limited guidance on optimal collaboration timeframes, Stephenson et al. (2024) suggest that therapy requires a minimum of 30 min, three times per week, to achieve meaningful outcomes, emphasising the importance of consistency and intensity. Hence, these variations of collaborative practices reported by the participants reflect differing levels of integration and scheduling of interdisciplinary teamwork within LSEN school environments.

Furthermore, these findings suggest a consistent pattern of collaborative engagement and indicate a foundational understanding and implementation of collaborative practices within LSEN school environments. They also provide insight into the existing collaborative landscape and highlight areas where further support may be needed.

Based on the researcher’s experience, the participants from the respective LSEN schools participating in the study typically designate the remaining weekdays (other than pull-out therapy times) for collaboration between teachers and SLTs. This collaboration occurs at scheduled times or as needed. However, further analysis is required to assess the adequacy and impact of these interactions. Furthermore, the variability in training, experience and collaboration frequency as presented in the results for Phase 1 suggests that while foundational practices are in place, gaps remain that may hinder optimal learner outcomes. Phase 2 (Interviews) will build on these insights by exploring participants’ lived experiences in greater detail. The ensuing discussions will then delve into these aspects.

### Phase 2

The qualitative findings are organised across ten identified themes, which are outlined in the results section. A comprehensive discussion of the four themes and examples of quotations from the participants, from Table 3, supported by pertinent literature, is presented below.

#### The role of speech-language therapists in learners with special educational need schools

Two participants noted that the interdisciplinary team offers academic support to learners with disabilities, including reader and scribe services. According to Malone et al. (2015) and Ramraj (2014), such support is often necessary for learners with visual impairments or intellectual disabilities who face challenges with reading, decoding of text or spelling. One participant shared:

‘And then our therapists … are all able to help with amanuensis. So, our therapists at exam times … they play the role of the readers and scribes for the learners …’ (P4, female, foundation phase teacher)

The findings highlight the collaboration of interdisciplinary teams in supporting teachers and learners in LSEN schools. Given that LSEN require tailored, integrated interventions to address complex learning and developmental barriers, the South African Education White Paper 6 introduced Individual Support Plans (ISPs) (also known as action plans) (De Villiers, 2012), which outline learner-specific needs, strategies and progress indicators. These plans highlight the importance of collaboration among professionals to meet these learners’ educational needs. Hence, the interdisciplinary team approach is essential. Archibald (2017) and Curro et al. (2022) further underscored the importance of teamwork in achieving educational goals for LSEN.

Two participants shared positive experiences regarding collaborative planning of learner activities with SLTs.

A participant remarked:

‘It is working together to get the best plan or the best activity for the benefit of the child eventually. And collaboration is very important in special schools [*LSEN schools*] because it is not a one man show, it is a team.’ (P2, female, foundation phase teacher)

Furthermore, seven participants reported positively on the unified visions and missions shared by teachers and SLTs for the learners. One participant responded:

‘On Mondays, we have learner discussions. So, then we sit together with our speech and language therapists … we discuss our learners, the learners they have, and then the progress that they have made, [*and*] the regression …’ (P4, female, foundation phase teacher)

Teachers’ experiences collaborating with SLTs in LSEN schools are mostly positive, aligning with Dillon et al. (2021), who noted that such collaboration enhances learner progress. Knight et al. (2016) found that working with SLTs improved children’s pronunciation, listening skills and speech-language delays, leading to therapy discontinuation for two children. Additionally, Mitchell (2017) reported that collaboration between SLTs and Grade 3 teachers resulted in positive vocabulary outcomes on three measures, reinforcing the benefits of collaborative planning and monitoring.

Three participants expressed positive views on collaboration between the teachers, SLTs and parents through consultations. One participant (P11) noted that therapists play a key role in supporting parents during meetings regarding the challenges and/or barriers that the learner experiences by stating:

‘What is good, though …, when we’ve got parent evenings, the therapist is … [*also present*]. So, she’ll join the parents with the teacher. And that’s very helpful to … put it forward [*to the parents*] that this is the problem [*challenges and/or barriers*] with the [*learner*], and that is very helpful so every term after the report, we will have a parent evening and the therapist joins. And that’s a good thing.’ (P11, female, foundation phase teacher)

Based on the findings, strengthening the active participation of families and all relevant stakeholders is essential during the current transition in departmental responsibilities. To ensure that children with disabilities are not ‘left behind’, both the Department of Social Development (DSD) and the Department of Basic Education (DBE) must establish structured meetings. These meetings should include parents, caregivers, educators, therapists, school management teams and district–level officials, and should focus on developing coordinated, cross–sectoral strategies for identifying, supporting and monitoring interventions for these children. The intention is to equip caregivers with clear, accessible information and ongoing guidance so that they are empowered to make informed, independent decisions aligned with the individual needs and best interests of their children (Karisa et al., 2022).

#### Collaboration benefits and practices

Pentek et al. (2018) found that consistent collaboration among SLTs, OTs and physiotherapists in LSEN schools led to positive outcomes, with 98% of learners making progress on their ISP goals. These findings underscore the importance of interdisciplinary teamwork and value of regular, coordinated teamwork in fostering meaningful progress for LSEN. In turn, it also supports Dillon et al. (2021), who emphasised that collaboration among parents, teachers and specialists is essential for achieving LSEN goals, such as enhancing communication skills and involving parents in goal selection and medical treatment for improved academic performance. Two participants shared that they received valuable guidance from SLTs classroom activities, as they sometimes felt uncertain about how to support learners with academic and communication challenges. One of the participant’s shared:

‘They [*SLTs*] will also give a lot of feedback with regards to, I see they [*learners*] can’t blend yet, or we have this new plan for their writing, and then I apply that [*the feedback from the SLT*] in my classroom …’ (P8, female, foundation phase teacher)

The findings underscore the significant role that SLTs play in guiding and coaching teachers within LSEN school environments. Mitchell (2017) highlights that SLTs often serve as trainers, particularly for Grade 1 and 2 teachers, by enhancing their understanding of oral language development and collaborative strategies. In the current study, teachers expressed interest in best practice models and suggested that classroom-based guidance from SLTs could improve their awareness of speech and language challenges. This aligns with the present findings, which demonstrate that SLTs are instrumental in equipping teachers with communication strategies, therapeutic insights, and classroom activities to support learners’ communication development.

Furthermore, four participants specifically noted that collaboration within the interdisciplinary team occurs through both formal and informal meetings. These meetings serve as platforms to discuss learner challenges, provide feedback and guidance to teachers, and coordinate appropriate interventions to support learners effectively. As one participant shared:

‘We speak a lot to our support teams. They are always [*present*] with learner discussions … They are there when we meet up with parents [*for parent meetings*] …’ (P5, female, foundation phase teacher)

In alignment with the findings, existing literature underscores the importance of SLTs prioritising collaboration with teachers particularly through regular meetings – as a foundation for effective interdisciplinary teamwork (Paul et al., 2018). In the current study, most participants reported collaborating directly with SLTs. Paul et al. (2018) claim that collaboration between teachers and SLTs promotes learner development and is essential for achieving optimal educational outcomes.

#### Teachers’ views on collaborating with speech-language therapists

Participants reported both positive and negative views on SLTs’ involvement in their classrooms. Five participants described meaningful support from SLTs, with one participant stating:

‘She [*SLT*] helped me a lot … I can go to her, and she helps me a lot.’ (P9, female, foundation phase teacher)

In contrast, four participants reported limited engagement, expressing a need for more support, as one participant noted:

‘I would love to have more input and more support on how to get them [*the learners*] to communicate, because at the moment [*currently*] I only have the visual schedule, and for me it’s not enough.’ (P2, female, foundation phase teacher)

Participants shared a range of perspectives and experiences regarding their collaborative involvement with SLTs in LSEN schools, which varied from active engagement to minimal participation. Positive feedback from participants aligns with findings by Mitchell (2017) and Olszewski et al. (2019), who emphasised the valuable contributions of SLTs in planning, promoting critical thinking, supporting study activities, and facilitating literacy development.

However, some participants expressed concern about the limited presence and involvement of SLTs within classrooms. Three participants perceived poor relationships in their collaborations with SLTs. A participant expressed the following:

‘I would say the relationship between teacher and therapist is not always as it should be because we do not get to spend a lot of time with [*the*] therapists at our school. Most of the time our therapist that is supposed to be here gets other duties, so we rarely see them in our classes taking the learners [*for therapy*].’ (P2, female, foundation phase teacher)

While teachers highlighted that SLTs are often given additional school duties, which creates competing priorities, and limits the time they can spend providing support to learners, this topic remains underexplored in current literature. Existing studies tend to address general barriers to collaboration, often overlooking SLTs’ limited classroom engagement and the specific dynamics of their interactions with teachers.

Strengthening professional relationships is essential for effective interdisciplinary collaboration. As Paul et al. (2018) suggest, successful partnerships often begin with individual teacher–SLT interactions and evolve as trust and rapport are built. Typically, SLTs initiate support, and as the benefits of collaboration become evident, the relationship deepens. Gallagher et al. (2019) and Olszewski et al. (2019) further emphasise that collaboration should involve equal participation and shared responsibilities, including joint planning and classroom-based support, to ensure cohesive service delivery.

Participants in the current study also shared practical insights into their experiences working with SLTs. Six participants reported that SLTs were frequently assigned additional duties beyond their core responsibilities. This negatively impacts SLTs’ capacity to provide consistent and effective support to teachers within the classroom setting. One participant reported:

‘I would also prefer if I could see my speech therapist more in a week and if she could provide me some assistance … Or maybe she’s observing a lesson of mine and telling me how can I improve or how do I or how must I talk to a child? Or how can I assist that child in that certain area.’ (P6, female, foundation phase teacher)

Additionally, five participants mentioned that they received limited support from SLTs. Some of the responses included:

‘I think I’ve seen the speech [*therapist*] once or twice the whole year. She just got in class and took the learners one time, and then the other time, she was there doing the activity with them. So it was only that twice, so I can’t really conclude. Just two visits for the whole year.’ (P10, female, foundation phase teacher)

Findings show that SLTs in schools are often overloaded with duties. Stephenson et al. (2024) noted that teachers observe SLTs struggling to address areas like reading because of large caseloads. Both studies highlight that SLTs are overburdened, limiting their ability to collaborate regularly with teachers and reducing the effectiveness of interdisciplinary support and classroom-based interventions.

Four participants additionally highlighted the limited number of SLTs employed in LSEN schools, which poses a challenge in managing classroom needs. As one participant noted:

‘As a school, we have one speech therapist … it is difficult to manage our classes.’ (P9, female, foundation phase teacher)

The findings highlight a growing shortage of SLTs in LSEN schools, worsened by rising learner enrolment, which challenges the sustainability of effective support services.

Two participants reported no challenges and expressed satisfaction with SLT services, as a participant noted:

‘Not [*any challenges*] that I can recall.’ (P5, female, foundation phase teacher)

However, the limited number of such responses is concerning.

Stephenson et al. (2024) identified several barriers to effective collaboration between teachers and SLTs, with one key concern being teachers’ resistance to SLT-led literacy support. This resistance often leads to confusion about each professional’s role. One participant’s (P3) response illustrated a general resistance to the provision of SLT support for learners, a sentiment that was not restricted to literacy-focused tasks. The participant stated:

‘I experienced that some teachers do not like other people interfering, if I can be honest. They would rather want to do it on their own and they do not want … help. So then at some stages I don’t think there’s any collaboration at all.’ (P3, female, foundation phase teacher)

Hence, the concern raised highlights the need for training on the importance of collaboration with SLTs and to clarify SLTs’ roles and address misconceptions that may hinder collaboration.

#### Teacher – Speech-language therapist collaboration needs

Participants were asked to propose practical solutions to the challenges they encountered. Several emphasised the need for teacher training, with four noting that many educators lack a clear understanding of the SLT’s role and scope of practice. One participant suggested:

‘We need training sessions on what speechies [*SLTs*] do and how they can support us to help the children, not just one child, but the whole class.’ (P7, female, foundation phase teacher)

These training needs align with Stephenson et al. (2024), who noted that limited understanding of SLTs’ roles and scope of practice among teachers can hinder collaboration. Paul et al. (2018) emphasised that in-service training is a practical approach to raising awareness of SLTs’ expertise and the value of collaborative practices in education. Additionally, two participants proposed solutions that the researcher identified as essential to strengthen relationships between teachers and SLTs. One participant remarked:

‘I think the relationship between therapists and teachers needs to be improved. Also getting to know the therapist better … there’s no time for that. We only see the therapists when we go home or when there is an event, but we do not really engage with them.’ (P3, female, foundation phase teacher)

Stephenson et al. (2024) emphasise that effective interdisciplinary collaboration, particularly among literacy-focused teachers, requires interprofessional practice, role clarity and a shared sense of belonging. Supporting this, two participants recommended reducing SLTs’ non-core duties to allow more time for direct learner support. One noted:

‘I … understand there’s a lot of kids [*learners in need of therapy*] and … we only have so many speech therapists, but I think it is possible to see the kids in groups or individually and get to all of them, … if your workload of other responsibilities is … less.’ (P2, female, foundation phase teacher)

The findings across the four main themes revealed that teachers in LSEN schools had varied experiences collaborating with SLTs. Many teachers appreciated the contributions of SLTs, particularly in learner support and planning. However, consistent collaboration was often disrupted by SLTs’ limited availability, largely because of high caseloads and involvement in non-core duties. Teachers also highlighted a lack of clarity regarding SLTs’ roles, especially in literacy support, and expressed the need for targeted in-service training to address this gap and recommended strengthening professional relationships to improve collaboration.

Despite these challenges, teachers recognised the value of interdisciplinary collaboration, particularly when SLTs were actively involved in learner discussions and classroom activities.

### Strengths of the study

This study offers valuable insights into teachers’ perceptions on collaboration with SLTs in a specific South African LSEN school context. By highlighting both the benefits and challenges of such collaboration, the study offers a balanced perspective on the dynamics at play. The findings have practical relevance for strengthening SLT–teacher partnerships, with the potential to enhance learner outcomes.

### Limitations of the study

This study’s limitations include a small, localised sample, which restricts the generalisability of findings to the broader South African context. As with most qualitative research, the data reflect individual experiences and cannot be broadly applied. Language barriers may have influenced participant responses, suggesting that the use of trained interpreters could have strengthened the data collection process. Additionally, the study highlights the need for further exploration into resource allocation and power dynamics within collaborative practices. Lastly, as the research focused solely on teachers’ perspectives, future studies should incorporate SLTs’ views to provide a more comprehensive understanding of collaboration in LSEN schools.

### Recommendations

To strengthen interdisciplinary collaboration in LSEN schools, the authors recommend improving communication between teachers and school management, alongside targeted training to clarify SLTs’ roles and manage teacher expectations. Outreach initiatives should be implemented to raise awareness of SLTs’ contributions among all stakeholders, including educators, administrators and policymakers. Sharing the study’s findings with these groups is essential to promote understanding and drive systemic change.

Further research exploring SLTs’ perspectives on collaboration is encouraged to provide a more comprehensive view and identify areas for improvement. Key recommendations include developing structured collaboration frameworks, scheduling regular interdisciplinary meetings and implementing joint training sessions. Evaluating the long-term impact of collaboration on learner outcomes is also advised. Expanding research into other educational contexts and providing evidence of effective collaborative service delivery will help inform best practices and guide future policy development.

## Conclusion

This study provides valuable insight into South African teachers’ perceptions regarding their collaboration with SLTs in schools catering for LSEN. By highlighting both the benefits and challenges inherent in such partnerships, this research underscores the critical role of effective interdisciplinary collaboration in education. As established, successful collaboration between professionals like SLTs and teachers is found on shared expertise and common goals to support learners (Banks, 2018), with a shared commitment being paramount to addressing diverse learner needs (Archibald, 2017; Mampane, 2016).

Guided by the chosen theoretical framework, our analysis consistently emphasised the intrinsic importance of teacher–SLT collaboration and affirmed the significant value that SLTs bring to supporting learners’ educational objectives. The findings illuminate key areas where interdisciplinary collaboration can be improved, paving the way for future research. Specifically, this study lays the groundwork for exploring SLTs’ perspectives and support needs, potentially leading to the development of researcher-led training initiatives designed to address identified collaborative challenges, deepen understanding of SLT roles and ultimately foster enhanced learner outcomes.
